# Harm reduction drug policy in Israel: what has been accomplished and what still needs to be done?

**DOI:** 10.1186/s13584-019-0343-3

**Published:** 2019-10-16

**Authors:** Hagit Bonny-Noach

**Affiliations:** 1 Department of Criminology, Faculty of Social Sciences and Humanities, Ariel University, 40700 Ariel, Israel; 2 Board member of the Israeli Society of Addiction Medicine (ILSAM), Ramat-Gan, & board member of the Israel National Anti-Doping Organization (INADO), Tel-Aviv, Israel

**Keywords:** Harm reduction, Drug policy, Substances use, Opioid maintenance therapy, Needle and syringe exchange programs, Israel

## Abstract

**Abstract:**

The leading formal drug policy in Israel is the traditional approach of abstinence, probation, and punitive measures based on three main pillars: Enforcement, Treatment and Rehabilitation, and Prevention. However, under the treatment pillar, Israel has adopted a number of harm reduction services, focused mostly on people who use heroin and people who inject drugs. These include Methadone Maintenance Treatment, Buprenorphine Maintenance Treatment, and Needle and Syringe Exchange Programs. More specialized services are designated mostly for people who use drugs, who frequent the largest open drug scene in Tel-Aviv. These include a health clinic, an emergency apartment for female addict sex-workers, and a ‘First Step’ center. Even so, the harm reduction approach has remained controversial, stigmatized, and is considered a sub-category for total-abstinence treatment in Israel. This paper follows the evolution of harm reduction interventions in Israel among people who use drugs and sheds light on the lack of a comprehensive, well-planned, formal national harm reduction drug policy. Additionally, this article expresses concern over the uncertain future of Israel’s comprehensive and balanced drug treatment policies caused by the structural changes in abolishing the Israel Anti-Drug Authority, the statutory authority and central body in Israel that promoted and coordinated all national policies related to treatment and harm reduction.

**Conclusions:**

Although it is a major challenge to translate worldwide evidence and research findings into action and social change, recommendations are offered to implement a comprehensive harm reduction drug policy led by a multidisciplinary group of policy-makers across all areas of drug policy. These focus on expanding and developing more services for Opioid Maintenance Therapy patients and people who inject drugs as well as a national effort to reduce high levels of stigma and discrimination against them, encompassing other common substances and focusing on populations such as adolescents and young adults that engage in other types of substance use such as cannabis, amphetamine-type stimulants, and hallucinogens.

## Background

The latest national epidemiological survey in Israel among adults aged 18–65 reported that 27% of the population used cannabis and 2% other illegal drugs in the past year, with 0.25% reporting heroin use during this period [1]. It is estimated that there are 15,000 to 25,000 people who use drugs (PWUD) in Israel [2, 3], although the exact number of people who inject drugs (PWID) is unknown [4], as is the exact HIV/AIDS prevalence among them. However, according to a database of PWID from the Ministry of Health’s (MOH) Department of Tuberculosis and AIDS (TB & AIDS), 260 AIDS and 997 HIV-Infected patients (1981–2017) were reported [5]. According to the MOH’s Department for the Treatment of Addictions (also called the Department for the Treatment of Substances Use), which has been collecting data on Opioid Maintenance Therapy (OMT) patients, 56% are Hepatitis C Virus (HCV), 18% are Hepatitis B Virus (HBV), and 5% are HIV-infected patients [6].

Among PWID, heroin is the most common injectable drug. However, there is an alarming spread of injectable new psychoactive substances (NPS) called *Hagigat* (i.e., ‘Celebration,’ the street name for increasingly common amphetamine/cathinone-type stimulants) and Ritalin (methylphenidate), mostly among PWID who gather in the largest open drug scene in Tel-Aviv [7–9].

*Drug policy* may range from “all activities related to illicit drugs” to “a set of principles or an ideology or system of laws, regulatory measures, courses of action and funding priorities that directs public action, governmental entity or its representatives concerning (illicit) psychoactive drugs (e.g., war on drugs, harm reduction, and more)” [10]. In accordance with the Single Convention on Narcotic Drugs [11], and the Israeli Dangerous Drugs Ordinance (New Version 5732, 1973), drugs were defined as a law enforcement issue and the leading formal drug policy in Israel became the traditional “war on drugs” approach that includes probation, punitive measures, and abstinence.

This approach is based on three main pillars: enforcement, treatment and rehabilitation, and prevention. More recently, prohibition-based drug policy has been challenged, debated, and questioned on multiple fronts for its harm, ineffectiveness, waste of resources, and, as a human rights violation, discrimination toward marginalized populations [12–15]. As such, there is an imperative for an updated drug policy [14, 16].

In Israel, the attitude of the establishment and general public has begun to soften. In parallel to the formal “war on drugs,” Israel has started to implement a “public health” approach, with authorities rolling out a ‘flexible’ drug policy. On the declarative *de jure* (legal) level, the war on drugs policy continues. However, the de facto (substantive) reality is focused mostly on drug dealers and less on users. As a result, in April 2019, Israel officially decriminalized adult use of cannabis.

Israel’s treatment and rehabilitation drug policy was the culmination of a long process, which began in the late 1970s as a response to lack of adequate care and solutions to the problem of people who use opioids (PWUO) [2, 17]. Heroin found its way onto the Israeli illegal drug market in around 1970, followed by a rapid increase in the number of heroin addicts. At that time, PWUO were offered barbiturates or transferred to closed wards in mental hospitals with other mental patients [17]. One of the first treatment options for PWUO began in Israel in 1975, with the establishment by MOH of two methadone maintenance treatment (MMT) centers and a drug-free rehabilitation center [18]. At that time, MMT centers were mainly following the model of Opioid Substitution Therapy (OST) – and less the OMT model.

Since the mid-1970s, Israel underwent an awakening of sorts in its need for a drug policy, when the number of drug users and amount of drugs seized by police began to increase. With media pressure, an inter-ministerial committee was formed in 1978 to formulate a comprehensive drug policy [17, 19]. In May 1983, the committee submitted its recommendations. During the 1980s, drug use in Israel evolved from a marginal concern to a social problem demanding a comprehensive solution. As a result, in 1985, MOH opened the Department for the Treatment of Substance Abuse [20]. The Ministry of Labor and Social Affairs (MOLSA) and the Ministry of Education (MOE) then established special departments for treating the problem of drug abuse. In addition, during the 1980s, the Israel Prison Service started providing treatment services for prisoners who used drugs, including MMT [3, 17, 21]. With 1987 defined as “The War on Drugs Year” by agencies and the Israeli government, media coverage of the problem of drug use in Israeli society was intensified in the public consciousness [22].

During those years, different ministries and non-governmental organizations (NGOs) attempted to offer solutions to the drug problem, but there was a lack of coordination among the various agencies as well as a lack of funding. Recognizing the need for a comprehensive and balanced approach, an inter-ministerial committee was appointed. Following the committee’s recommendations, the Israel Anti-Drug Authority (IADA) was established in 1988 as a statutory corporation [2]. The establishment of IADA was part of Israel’s efforts to comply with the 1971 UN Convention on Psychotropic Substances (Art.6), which called for a national anti-drug authority. IADA’s establishment also facilitated compliance with all other UN conventions in all areas of demand and supply reduction [3]. IADA, under the authority of the Office of the Prime Minister, was the central body promoting inter-ministerial and inter-institutional cooperation and activities as well as formulating all national policies, including those related to treatment and rehabilitation.

With the need for comprehensive treatment models, IADA started to coordinate between MOH and MOLSA, which are jointly responsible for the treatment and rehabilitation of PWUD, but have different treatment perspectives. MOH considers addiction mainly as a health problem and operates medical and harm reduction treatments, while MOLSA views addiction as a social psychological problem and operates cognitive behavioral abstinence treatments [23, 24].

IADA also initiated services and programs against drug abuse and for PWUD, encouraging and funding research on data-based policies [21, 25, 26]. In 1989, the Special Committee on Drug and Alcohol Abuse (SCDAA) in the Israeli Knesset (parliament) was established. SCDAA supervised all authorities that deal with drug abuse [27]. In parallel, the penalties imposed on drug offenses (but not for users) in the Dangerous Drugs Ordinance were increased. From the 1990s, various programs for treatment and rehabilitations services in Israel were established, which offered a myriad of treatment solutions [2, 3].

### Lack of comprehensive harm reduction drug policy

*Harm reduction* as a drug policy can be defined as policies, programs, and practices that aim primarily to reduce the adverse health, social, and economic consequences of the use of legal and illegal psychoactive drugs – without necessarily reducing drug consumption [28]. Harm reduction provides an alternative to the classic criminalization option [29]. It has a human rights agenda in bringing effective treatment to traditionally marginalized groups. However, it is confronted by complex ethical dilemmas due to its non-judgmental approach toward users who may pose threats to themselves and their communities [30]. Historically, harm reduction has been overwhelmingly associated with interventions aimed to reduce the health harms associated with the injection of opioids such as Opioid Maintenance Therapy (OMT), Needle and Syringe Exchange Programs (NSEP), and safer injecting facilities. Most interventions focus on the injection of opioids, although harm reduction applies to all types of substances and drug use [31]. In fact, during the past three decades, harm reduction has emerged as a stable doctrine in health-related drug policy [32, 33]. Many governmental agencies and NGOs support the promotion of harm reduction policy [4, 31]. Western Europe is a leading supporter of harm reduction policy and practice that is now positioned as part of the mainstream policy response to drug use [34].

Israel is influenced by the activity of many agencies in the field of harm reduction, mostly agencies in Western Europe [2, 3]. More recently, treatment professionals and policymakers mostly from IADA and MOH have expressed interest in harm reduction approaches [2, 3, 9, 35]. Israel has started to establish some harm reduction interventions, mostly for PWUO and mostly by MOH and IADA [3]. According to the Global State of Harm Reduction (GSHR), no explicit supportive documentary reference to harm reduction in Israeli national policy existed until 2016 [4]. However, according to the newest GSHR published at the end of 2018, Israel has started to explicitly make supportive reference to harm reduction in national policy documents [36]. Although OMT has existed in Israel since the mid-1970s, it is not considered a classic harm reduction strategy. National policy documents refer to OMT as a “substitution” or “long term medication care.” In those documents, only NSEP is defined under the title of ‘classic’ harm reduction intervention [35, 37].

As noted, IADA and MOH are explicitly supportive of a few harm reduction programs. However, on the declarative level, the main drug policy still supports total abstinence. Harm reduction interventions thus remain the last resort for PWUD. The harm reduction policy of Israel was never clearly planned, balanced or comprehensive. In fact, lately there is concern over the uncertain future of all policies related to treatment, including the harm reduction policy. In February 2018, the Knesset abolished the Israel Anti-Drug Authority Law. The IADA is no longer a statutory corporation, but is rather to become part of the Ministry of Public Security (MOPS) and will be renamed the Authority for Combating Violence, Drugs and Alcohol. MOPS is responsible for law enforcement and security, and so the future of comprehensive and balanced approaches to drug treatment policy is very much a matter of concern. The new authority will probably focus more on enforcement and prevention pillars at the expense of treatment and rehabilitation pillars. This may well affect the continuity of promoting coordination of government ministries and NGOs in formulating national policies related to treatment and rehabilitation and harm reduction.

### Opioid maintenance therapy (OMT): methadone maintenance treatment (MMT) and buprenorphine maintenance treatment (BMT)

Israel is part of the first wave of countries to institute MMT for PWUO, a treatment method that started in the mid-1970s. Opiate addiction was then considered a chronic condition, and, therefore, the main goal was not abstinence, but rather trying to stabilize PWUO and expose them to life without crime [17, 18]. In Israel, in the mid-1980s, a great deal of controversy emerged over the role of MMT. A reaction started to support drug-free treatment. Policy makers took the view that addiction was not necessarily a chronic constant condition, deviating from the conventional notion of the time that “once a drug addict, always a drug addict.” That is, a policy position started to develop around the claim that users could be fully rehabilitated to a completely drug-free life. As a result, during the late 1980s, IADA recommended reducing MMT distribution to PWUO and expanded and supported the establishment of a variety of total abstinence treatment options such as therapeutic communities [17]. Additionally, MMT treatment was changed from drug substitution only to an integrative treatment, including psycho-social support from multi-disciplinary professionals [2, 20, 38].

During the 1990s, in accordance with the drug-free policy, MMT was pushed to the margins of the therapeutic system, its professional status and budget neglected [21]. In this period, the regulations prohibited privately run MMTs (except for a single private clinic) to continue and their operation was exclusively in the hands of MOH [3]. Until recently, the amount of methadone approved by the MOH for distribution was limited and PWUD who wanted to receive MMT would have to wait sometimes over a year [39]. Currently, there are no waiting lists for any of the MMTs in the country. Most research on Israeli PWUD who are MMT patients confirms the advantages associated with MMT: reduction of opiate abuse, decrease in death rate, and lowering the risk of other complications [40, 41]. Even so, the stigma attached to MMT is very common, even among addiction facility professionals in the social services departments [42]. The criticism and stigma accorded MMT also came from a large group in Israel of ex-PWUD, members of the Narcotics Anonymous (NA). The NA concept of abstinence can be controversial as methadone is considered a psychoactive substance similar to street drugs. For most NA members, individuals who consume methadone are actively addicted and thus a threat to the NA member philosophy [44]. As a result, most NA members refrain from contact with MMT patients, derogating them as simply lacking willpower.

Buprenorphine Maintenance Treatment (BMT) has been available in Israel since 2002 [45]. In 2013, buprenorphine (Subxone) was included in the health-drug basket, the first substitute for addictions of its kind [46]. In recent years, BMT has been allocated higher priority than MMT by MOH [47]. MOH now claims that buprenorphine, as a partial agonist, is safer than the full agonist, methadone. Buprenorphine is suggested as an opioid replacement therapy during pregnancy, causing fewer neonatal abstinence syndrome symptoms than methadone, with a lower level of dependence and tolerance. With longer duration of action and lower risk of fatal overdosing, PWUDs can be treated in their community and no longer require necessary daily clinic visits [39, 48]. Contrary to MMT, BMT is available in hospitals and a small number of private clinics who received appropriate licensing and are supervised by MOH [3]. Despite the relatively high price of the treatment in private clinics, most young PWUD prefer to receive BMT there due to limited medical supervision (such as random urine tests) and minimal or absent psycho-social support [23].

With changes to the OST model, the OMT is the currently preferred model, one that includes medical and psycho-social interventions encompassing harm reduction interventions such as identification, prevention, and referral to treatment of infectious diseases.

The number of OMT patients in the country has increased, and currently almost a third of all known PWUO (more than 4000 patients every year) receive OMT treatment [49, 50]. MOH declared that OMT is an effective and safe way to treat PWUO who want to stop using opiates, greatly reducing the direct and indirect harms of addiction [38]. Even so, OMT remains a marginal part of the comprehensive drug-free treatment system, the last choice of treatment for PWUD in Israel and given to PWUD only as a last resort after total abstinence treatments [3]. In MOH formal documents, it was reported that there are only 12 public units and 6 private clinics for OMT across the country. Most of the units and services in Israel are for abstinence patients who receive treatment in MOLSA units [50]. The exception is in Israel’s prison system, where there was 40 OMT for 600 clients [51].

In recent years, the formal policy documents from IADA and MOH offer PWUD two treatment tracks: 1) abstinence and 2) MMT and BMT. As noted, the terminology still considers the latter as inferior to the former. For example, in 2011, IADA, MOH and MOLSA official documents noted that the target population for long term medication treatment using MMT and BMT are “PWUO who have not been successful in previous treatments in the complete detoxification path, and have reverted to drug use and non-normative and dysfunctional behavior that accompanies use” [37]. The terminology was changed in the 2015 revision to “PWUD who have not been able to completely quit after repeated attempts” [50]. This notion of OMT as a second choice for PWUD continues into MOH’s 2016 annual report that stated: “OMT was designed to provide a solution for those who have failed in their attempts to complete rehab without medication due to a severe addiction disease. Programs through maintained medication such as methadone or Subutex and Suboxone accompanied by psychosocial therapy are offered” ([49], p 12).

### Needle and syringe exchange programs (NSEP) and services

According to national data from MOH’s Department of TB & AIDS, PWID are one of the high risk groups for HIV/AIDS infection in Israel. In the late-1990s and beginning of the twenty-first century, an increase of HIV infections among drug users was noted, especially among new immigrants [52, 53]. New immigrants from the former Soviet Union (FSU) brought their heroin injecting patterns with them [54]. As AIDS is considered a greater threat to health than the dangers of drug use, the TB & AIDS Department, in collaboration with IADA and the Jerusalem Methadone Center, initiated the first experimental project of NSEP in Jerusalem in order to decrease the extent of needle-transmitted infections [52, 55, 56]. During 2004 and 2005, NSEP was initiated in three major cities and 450 PWID were included in this program. The justification for NSEP was the health risk factor for needle-transmitted infections such as HIV, Hepatitis B Virus (HBV), and Hepatitis C Virus (HCV).

PWID tend to be characterized by behavioral patterns including shared use of needles and paraphernalia and unprotected intercourse [57]. In 2007, the Yizhar program was established by the Public Health Association, an NGO created by MOH, which also operates some of the public MMT centers in Israel. Yizhar is supervised by MOH’s TB & AIDS Department, IADA’s Treatment of Substance Abuse Departments, and the NGO, the Israeli AIDS Task Force. Yizhar operates NSEP in five cities with a base of professionals, although it relies mainly on volunteers. These NSEP centers provide additional services such as paraphernalia, condoms, warm beverages, food, clothes and shower facilities [53, 58]. From 2008 to 2012, about 4000 PWID were treated in these centers and some 800 were referred to detox or OST [47]. In addition, Yizhar volunteers wander the streets where the hard-to-reach user population gathers, especially at nights, in order to distribute syringes to them in the field. In 2015, 214,777 syringes were distributed [59, 60]. HIV diagnoses among PWID declined in the absolute numbers of HIV cases, from 70 cases in 2004 to 42 in 2008, and these lower numbers remained constant until 2011 [53]. However, a year later, there was a sharp increase in new HIV cases [8, 61, 62], mostly among PWID that gathered in Tel-Aviv’s open drug scene. These outbreaks were associated with changes in injectable drugs – from heroin to the cheaper *Hagigat* that requires many more daily injections and does not require sterilizing cooking and boiling. Only pre-injection melting is needed due to the high solubility of the new compounds [9]. In the following years, the number of new HIV cases among PWID has decreased [62].

NSEP in Israel is supported by governmental agencies and public health associations. Studies on NSEP show its positive effect on preventing spread of infectious diseases and reducing rates of HIV [33, 63]. However, NSEP suffers from lack of funding, and is based mainly on volunteer staff.

The call for more harm reduction services of OST and NSEP was emphasized in the introductory section of MOH’s Department for the Treatment of Substances Use annual report for 2016. The adoption of the main drug policy recommendations of the United Nations General Assembly (UNGASS) from 2016 was mentioned. It was also suggested to expand OST and NSEP for PWUO, along with development of harm reduction programs such as Naltrexone for prevention of overdose death [49].

### Special services for PWID in Tel-Aviv’s largest open drug scene

Open drug scenes are defined as settings where public use and trade of drugs occurs [64]. They exist in several cities in Israel, the largest of which is located in the old central bus station in Tel Aviv, which began to take shape in the mid-2000s. Most homeless PWID are found in this area [7, 8, 65]. The PWID in the open drug scene who inject heroin, *Hagigat*, Ritalin, and other mixed substances are considered socially inferior and marginal [7, 8]. In this area, the authorities and NGOs established a few harm reduction interventions. These include the Levinsky Clinic, established in 2002 by the District Health Office of Tel Aviv as a treatment and harm reduction community clinic for sex workers and prevention of sexually transmitted diseases. The clinic offers voluntary medical care for the addict population that continues to gather in the area. Another service is the First Step Center (FSC) that was founded in 2006 by IADA in cooperation with MOH. The center refers PWID to needed services, including detoxification, OMT, clinics for treating STDs, etc. In 2007, the Yizhar NSEP program was established and the Tel Aviv unit was also located in the FSC, providing PWID with resources such as showers, clothes, condoms, snacks and hot drinks, or just a chat with professionals and volunteers at the center. Once a week, it functions as a harm reduction center for women only [8, 66]. In 2009, an emergency apartment called *Saleet* was established for addicted women engaged in prostitution and living on the street.

### Harm reduction among youth and young-adult populations

Policy makers have recently adopted two approaches among youth and young adult populations based on harm reduction. The first is a comprehensive alcohol consumption strategy such as “Drink responsibly.” This takes into account the harm reduction pillar in addition to the prevention, treatment and law enforcement pillars [67]. The second is the harm reduction approach for young-adult backpackers who use drugs. This includes providing tips for backpackers and information in case of emergencies such as acute psychosis due to substance abuse. In addition, an open house information resource center called the ‘Israeli Warm Home’ was set up in India in 2003. It was established as a first response site for those negatively affected by drug use [27, 68, 69]. These initiatives are intended to reduce drug abuse and provide assistance to young backpackers far from home. Protecting young adults from drug-related harm, such as young Israeli backpackers, highlights how much can be accomplished when policymakers and the public approach harm reduction as a net benefit to their own children and peers [70].

In addition, new volunteer initiatives for young-adults were set up to provide safety information and consulting as well as safe zone at raves (i.e. large techno music parties) for people who use ATS and hallucinogenic substances. One of these projects is called “Good People” and was initiated by Elem, a youth-in-distress non-governmental organization. Their volunteers identify young people in crisis due to psychoactive substances at popular events like raves. They stay with the individual to provide psychological aid and support. In 2017, the project team reported having treated about 200 emergency cases [71]. Recently, following the deaths of young people from the LGBT community related to drug use, the LGBT community embarked on a cooperative initiative with city health and welfare representatives to develop harm reduction interventions.

## Conclusions: what more needs to be done?

Israel was an innovator in harm reduction services, such as MMT as an acceptable treatment form, in the mid-1970s. During the intervening years, other harm reduction services developed, mostly for PWUO, such as BMT, NESP, and specialized services for PWUD in the largest open drug scene in Tel-Aviv. Even so, and despite some positive statements on harm reduction policy from national agencies such as MOH and IADA, there is still criticism and controversy regarding this policy. Although it is a major challenge to translate worldwide evidence and research findings into action and social change, and it is also challenging to translate the evidence into the local reality, Israel should adopt and implement a comprehensive harm reduction policy led by a multidisciplinary group of policy-maker representatives from all the relevant ministries. Ultimately, society’s drug problems cannot be solved by a single government agency alone such as MOH, almost the only agency that deals with harm reduction policy. Rather, harm reduction approaches and principles should be integrated across all areas of drug policy. They should be applied to all services that work with people who use drugs, with the understanding, support, and collaboration of law enforcement agencies [31]. Parallel recommendations to public policies and collaborations across sectors and levels of government can be found in the “Health in All Policies” (HiAP) approach from WHO. HiAP systematically takes into account the health implications of decisions, seeks synergies, and avoids harmful health impacts in order to improve population health and health equity. It can provide a framework for regulation and practical tools that combine health, social and equity goals with economic development, and manage conflicts of interest transparently [72].

The lack of comprehensive drug policy in Israel is noted by some addiction professionals, who complain about insufficient resources to make the existing treatment system accessible to needy population groups. The need for a common interface between the two bodies responsible for addiction treatment frameworks, MOH and MOLSA, is also noted [23]. As the two major ministries still have different policy approaches to addiction: MOH – public heath approaches and harm reduction and MOLSA – total abstinence. However, the latter does not fit the needs of a wide part of the PWUD. Both ministries should thus coordinate their service planning and agree on a comprehensive treatment-harm reduction national policy.

Israel has implemented some of the interventions according to the international standards for a comprehensive package of services for PWID endorsed by the World Health Organization (WHO), the United Nations Office on Drugs and Crime (UNODC), and the Joint United Nations Program on HIV/AIDS (UNAIDS) [33] as well as the U.S. President’s Emergency Plan for AIDS Relief (PEPFAR) [73], yet more needs to be done.

There is a need for less strict conditions for OMT patients then currently exist [38]. Also, the aging of OMT patients raises the need to build rehabilitation services that are suitable for the needs of these patients, with an emphasis on occupational rehabilitation [23]. The small-scale (only in five cities) and NGO-driven NSEP should be improved. Underfunded, it relies mainly on volunteers and lacks strong political support. More professionals such as nurses and medical supervision in treatment centers are also critical. In any case, in the absence of health personnel, the development of an organized network of volunteers, including expert volunteers who receive better training, can be helpful. NSEP should provide more equipment such as sterile water, drug checking, and equipment kits for PWID. Additionally, NSEP and FSC service hours should be extended, as revealed in a recent survey of PWID in Israel [8]. Also, Israel should adopt more radical harm reduction interventions for PWID such as providing Naltrexone to prevent death by overdose, consumption rooms, and heroin-assisted treatment (HAT) [8]. The call for some of these recommendations, as well as a comprehensive national policy, appears in the introductory section of MOH’s Department for the Treatment of Substances Use annual report for 2016 [49]. However, in the annual report of 2017, these recommendations for expansion of OMT and NSEP and provision of Naltrexone were omitted [6].

As noted, harm reduction services are still considered a last resort for PWUO after abstinence treatments. This is so, even though almost a third of PWUO receive OMT and data confirms the success of harm reduction services in Israel and other countries [33]. Indeed, there is persistent misunderstanding and denial of the needs of PWID by the authorities and the Israeli public [7, 8]. Thus, there is a need for promoting increased public awareness of Substance Insecurity, defined as the uncertain availability of quality substances (or their substitutes) and ability to acquire them and safe injection equipment in socially acceptable (or not) ways [8].

In addition, it is important to draw attention to unique harm reduction interventions for individuals with HCV, HBV, and HIV among PWID. Even though 2012 and 2013 saw an increase in new HIV cases due to injection of *Hagigat,* an amphetamine-type stimulant (ATS), in the open drug scene [9, 61, 62], the harm reduction policy and services did not change with these circumstances, and ATS harm reduction strategies and services are not readily available. In fact, the drug market dynamic is continuously changing as new substances and new forms of consumption, along with related behaviors, are introduced into the drug-using community. Harm reduction services should be updated to stay current with new trends and adapt relevant responses and services.

A national effort to reduce high levels of stigma and discrimination against OMT patients and PWID is an important and necessary undertaking. Harm reduction awareness should target professionals and public alike. The involvement of more civil society organizations (CSOs) [31] and relocation of OMT units from mostly industrial urban settings to ‘normative locations’ such as hospitals or public clinics, of which only a few OMT units serve as a precedent, may assist awareness campaigns. Educational intervention, especially among social services department personnel, may benefit people who use opioids and improve the overall quality of treatment for opioid addiction in Israel [42].

In recent years, MOH and MOLSA identified a change in the profile of PWUD in Israel. PWUO are aging and, therefore, need new facilities for older OMT patients such as home visits and home-delivery of medications – even as more young adults with higher socio-economic status are known to use cannabis, NPS, prescription drugs, and other illicit drugs [16, 73]. However, harm reduction approaches, which have some legitimacy in specific domains such as opioid addiction and among PWID, are generally not considered acceptable by the authorities and professionals for non-opioid and non-injecting drug use, especially as an approach for adolescents and young adults engaging in other types of substances use.

Of course, most substance users are young adults who favor cannabis [1]. Indeed, recent changes in the legal status of cannabis in Israel from probation to decriminalization are potentially transformative. However, they do not alter the fact that (except for the enforcement pillar) no clear comprehensive enforcement, treatment, prevention, and harm reduction drug policy plan for cannabis exists. One harm reduction intervention recommendation is to provide appropriate information about safer methods of drug use. Medical cannabis patients and young adult recreational users share information and tips on how to avoid harmful cannabis use in informal groups and online cannabis chat forums. However, this should not be mistaken for an official and formal harm reduction plan.

Additionally, more harm reduction measures should be taken for youth and young-adult populations. New treatment projects for people who use ATS and hallucinogenic substances at mass gatherings such as raves [71] should be formalized with supervision by the authorities and policy makers, as is typical with existing comprehensive alcohol consumption strategy [67] and for backpackers [68, 69]. In fact, these examples highlight how much can be accomplished when policymakers and the public approach harm reduction as a net benefit to their own children and peers [70]. Nevertheless, there is still more to be done to arrive at a comprehensive harm reduction policy to reduce high risk health behaviors in young adults and other populations.

In conclusion, a gap exists between comprehensive harm reduction policies as outlined in international documents and research findings and as they are actually implemented in Israel by governmental agencies. A multisector response is required to ameliorate the harms associated with drug addiction in the country.

## Data Availability

The data used in this paper is based on official documents, protocols, and reports available in IADA and MOH archives. Most are available on IADA and MOH webpages (links are available in the reference section).

## Abbreviations

ATS: Amphetamine-Type Stimulants
BMT: Buprenorphine Maintenance Treatment
CSOs: Civil Society Organizations
EMCDDA: European Monitoring Centre for Drugs and Drug Addiction
FSC: First Step Center
FSU: Former Soviet Union
GSHR: Global State of Harm Reduction
HAT: Heroin-Assisted Treatment
HBV: Hepatitis B Virus
HCV: Hepatitis C Virus
HiAP: Health in All Policies
HIV: Human Immunodeficiency
IADA: Israel Anti-Drug Authority
IADAV: Israel Anti-Drug, Alcohol and Violence Authority
IDPC: International Drug Policy Consortium
MMT: Methadone Maintenance Treatment
MOE: Ministry of Education
MOH: Ministry of Health
MOLSA: Ministry of Labor and Social Affairs
MOPS: Ministry of Public Security
NA: Narcotics Anonymous
NGO: Non-Governmental Organization
NPS: New Psychoactive Substances
NSEP: Needle and Syringe Exchange Programs
OMT: Opioid Maintenance Therapy
OST: Opioid Substitution Therapy
PEPFAR: President’s Emergency Plan for AIDS Relief
PWID: People Who Inject Drugs
PWUD: People Who Use Drugs
PWUO: People Who Use Opioids
SCDAA: Special Committee on Drug and Alcohol Abuse
TB & AIDS: Department of Tuberculosis and AIDS
UNAIDS: United Nations Program on HIV/AIDS
UNGASS: United Nations General Assembly
UNODC: United Nations Office on Drugs and Crime
WHO: World Health Organization
